# Outcomes of Persistent Microvascular Inflammation in Repeated Kidney Allograft Biopsies

**DOI:** 10.1111/ctr.70419

**Published:** 2025-12-11

**Authors:** Sandesh Parajuli, Adam Bregman, Emily E. Zona, Megan Sokup, Neetika Garg, Weixiong Zhong, Didier Mandelbrot

**Affiliations:** ^1^ Division of Nephrology Department of Medicine University of Wisconsin School of Medicine and Public Health Madison Wisconsin USA; ^2^ Department of Pathology University of Wisconsin School of Medicine and Public Health Madison Wisconsin USA

**Keywords:** kidney transplant, outcomes, persistent, microvascular inflammation

## Abstract

**Background:**

Microvascular inflammation (MVI) with sum glomerulitis and peritubular capillaritis (g+ptc) ≥ 2 is an integral component of kidney allograft antibody‐mediated rejection (AMR). It is unclear what the outcomes are among those with persistent MVI, despite treatment.

**Methods:**

We included all kidney transplant recipients (KTRs) with persistent MVI ≥ 2 on first and second allograft biopsies who had third biopsies within 2 years of the first biopsy. KTRs were categorized into two groups, MVI (+) and MVI (−) on third biopsy. Risk factors for persistent MVI ≥ 2 on third biopsy, and graft survival based on MVI (+) and MVI (−) at last follow‐up were outcomes of interest.

**Results:**

A total of 108 KTRs transplanted between 2013 and 2022 fulfilled our selection criteria, 75 (69%) were MVI (+) and 33 (31%) MVI (−). Most baseline characteristics were similar between the groups. In Cox regression analysis, none of the commonly assessed baseline characteristics, Banff scores, or DSA status at first or second biopsy were associated with persistent MVI on the third biopsy. Also, in Cox regression analysis, after adjusting for various characteristics, persistent MVI on third biopsy was not associated with increased or decreased risk for uncensored graft failure (aHR: 0.55, 95% CI: 0.23–1.29; *p* = 0.17).

**Conclusion:**

The lack of difference in graft outcomes between the AMR patients who were MVI (+) versus MVI (−) on the third biopsy suggests that subsequent response to AMR treatment is less important for prognosis than the initial development of AMR. This reinforces the importance of the prevention of rejection.

**Abbreviations**
aHR: adjusted hazard ratio
AMR: antibody‐mediated rejection
cg: transplant glomerulopathy
DCGF: death censored graft failure
dd‐cfDNA: donor‐derived cell‐free DNA
DGF: delayed graft function
DSA: donor‐specific antibodies
eGFR: estimated glomerular filtration rate
g: glomerulitis
GF: graft failure
HLA: human leukocyte antigen
KTRs: kidney transplant recipients
MFI: mean fluorescent intensity
MVI: microvascular inflammation
ptc: peritubular capillaritis

## 1 Introduction

Antibody‐mediated rejection (AMR) is a significant contributor to late kidney allograft failure [1]. Microvascular inflammation (MVI) is an integral component for the diagnosis of AMR [2]. The Banff classification incorporates a scoring system that combines glomerulitis (g) and peritubular capillaritis (ptc) into a total MVI score ranging from 0 to 6, provided that glomerulonephritis and/or tubulo‐interstitial inflammation are absent [2]. MVI has long been recognized as a hallmark of AMR. MVI, along with detectable donor‐specific antibodies (DSA) and/or peritubular capillary C4d staining, constitutes a key diagnostic criterion for the diagnosis of AMR [3]. However, the updated Banff 2022 now includes two additional subcategories: “Probable AMR,” characterized by MVI below defined diagnostic thresholds in DSA‐positive patients, and “MVI, DSA‐negative, C4d‐negative.” Both were categorized as “No rejection” in the previous Banff 2017/2019 scheme [4]. Patients with MVI, in the absence of DSA or C4d, are at increased risk for developing classic AMR and progressive transplant glomerulopathy, ultimately leading to graft failure [5]. Among all the criteria of AMR, MVI has the highest correlation with graft failure or decline in estimated glomerular filtration rate (eGFR) in the following 2–5 years [5].

There are no effective treatments for C4d and DSA‐negative MVI. For many years, our program has chosen to treat patients with MVI ≥ 2 as having AMR. Also, in our program, all patients with the diagnosis and treatment of AMR (including isolated MVI ≥ 2) underwent follow‐up biopsy after approximately 12 weeks of treatment. It is not uncommon for patients to have persistent MVI despite aggressive treatment. The negative impact of persistent MVI on follow up biopsy (second biopsy) after treatment of AMR has been reported in multiple studies [6, 7, 8, 9]. However, to the best of our knowledge, no data reports outcomes of persistent MVI beyond second biopsies. Here we report our experience with patients having persistent MVI ≥ 2 on the follow‐up biopsy (second biopsy), who also had undergone further follow‐up biopsy as a third biopsy, after the treatments. Patients were categorized based on the persistent MVI ≥ 2 (MVI (+) or resolution on the third biopsy (MVI (−). We hypothesized that recipients with MVI (+) on the third biopsy would have detrimental outcomes.

## 2 Methods

This was a single‐center study conducted at the University of Wisconsin that included all adult kidney transplants between January 2013 and June 2023. We included only recipients who had at least two follow‐up biopsies after the index biopsy (first biopsy) with MVI ≥ 2, and had persistent MVI ≥ 2 in the second biopsy. All three biopsies were performed within two years. Recipients were categorized into two groups based on the persistent MVI ≥ 2 ((MVI (+)) or MVI <2 ((MVI−)). We excluded recipients who did not have at least 3 biopsies or did not have two consecutive biopsies with MVI ≥ 2.

The outcome of interest was to assess the risk factors for the persistent MVI ≥ 2 on the third biopsy. Further, we assessed the risk of uncensored graft failure (GF) and death‐censored graft failure (DCGF) based on the persistent MVI ≥ 2. We looked at the graft function assessed by serum creatinine and estimated eGFR rate utilizing the 2021 CKD‐EPI equation eGFR. GF was defined as all causes of graft failure, including death. DCGF was defined as graft failure requiring chronic dialysis or re‐transplantation. The patients were followed until the GF or DCGF or the end of the data analysis in January 2025.

This study was approved by the University of Wisconsin Health Sciences Institutional Review Board (IRB protocol number: 2014‐1072) and adhered to the Declaration of Helsinki. The clinical and research activities reported were consistent with the Principles of the Declaration of Istanbul on Organ Trafficking and Transplant Tourism. Due to the nature of this study, informed consent specific to this research was not obtained from patients.

### 2.1 Immunosuppressive Protocols

The standard protocol for most kidney transplant recipients (KTRs) at our institution involved induction therapy using either a depleting agent (anti‐thymocyte globulin or alemtuzumab) or a non‐depleting agent (basiliximab). This was followed by a maintenance immunosuppressive regimen of tacrolimus and mycophenolic acid, with or without prednisone. The choice between depleting and non‐depleting agents was based on immunological risk factors. Depleting agents were preferred for patients with pre‐transplant DSA, glomerulonephritis as the cause of kidney disease, and those who will undergo early steroid withdrawal [10].

#### 2.1.1 Kidney Allograft Biopsy

Biopsies were performed mainly for cause, such as an unexplained increase in serum creatinine or proteinuria. Protocol biopsies were performed at 3 and 12 months for patients with pretransplant DSA, or for those who developed de novo DSA [11]. All recipients treated for MVI ≥ 2 had follow‐up biopsies within 12 ± 2 weeks after treatment. It has been our practice to perform follow‐up biopsies on all patients treated for MVI, even after the initial follow‐up biopsies, to assess the resolution of MVI.

#### 2.1.2 Rejection Protocols

The treatment for allograft rejection varied based on the severity and timing of AMR as previously described [12]. Briefly, AMR within 3 months posttransplant was treated with dexamethasone bolus, plasmapheresis, and intravenous immunoglobulin (IVIG). The treatment regimen for episodes occurring more than 3 months posttransplant included a dexamethasone bolus with taper, IVIG, plus/minus rituximab, depending on the severity of the rejection. As above, we have treated MVI ≥ 2 with a similar regimen as AMR during the study period. If recipients were not on steroids before AMR, low‐dose prednisone was continued after the management of AMR. Although not formally protocolized, some recipients with persistent MVI also had an increase in the baseline immunosuppression by adding belatacept, and were maintained on quadruple immunosuppression based on the treating provider's discretion.

### 2.2 Statistical Analysis

Continuous data were compared using Student's *t*‐test or the Wilcoxon rank‐sum test, as appropriate, while categorical data were analyzed using Fisher's exact test or chi‐square test. *p* values ≤ 0.05 were considered statistically significant. Factors associated with persistent MVI ≥ 2 on the third biopsy, along with the risk for GF and DCGF for each risk factor, were computed using both univariate and multivariate analyses. Due to the limited sample size of outcomes of interest, only some pertinent risk factors from the baseline demographics and first or subsequent biopsies were included in the univariate analysis. Only factors with *p* ≥ 0.10 in the univariate analysis were included in the multivariate analysis. Kaplan–Meier analyses were performed for GF and DCGF from the first biopsy and the third biopsy.

## 3 Results

A total of 108 KTRs fulfilled our selection criteria (Table 1). A total of 75 (69%) were in the MVI (+) group, and 33 (31%) were in the MVI (−). Of these 33 patients with MVI (−), 24 had an MVI score of 0, and the remaining 9 had an MVI score of 1 (3 with g1, and 6 with ptc 1). Most of the commonly assessed baseline characteristics were statistically similar between the two groups, except that the rate of delayed graft function was significantly higher in the resolution of MVI group.

**TABLE 1:** Baseline characteristics.

Variables	Total (*n* = 108)	Persistent MVI on the third biopsy (*n* = 75)	Resolution of MVI on third biopsy (*n* = 33)	*p*
Mean age at transplant (yrs)	48.9 ± 15.5	48.8 ± 14.5	49.1 ± 17.6	0.93
Male (%)	74 (69)	52 (69)	22 (67)	0.78
White (%)	81 (76)	58 (77)	23 (72)	0.55
Recipient's BMI (kg/m^2^)	27.1 ± 5.2	26. 7 ± 4.9	28.0 ± 5.9	0.29
ESKD causes (%)				0.70
Diabetes	20 (19)	15 (20)	5 (15)	
Hypertension	10 (9)	8 (11)	2 (6)	
Glomerulonephritis	38 (35)	26 (35)	12 (36)	
Polycystic kidney disease	16 (15)	9 (12)	7 (21)	
Other/unknown	24 (22)	17 (23)	7 (21)	
Previous transplant (%)	43 (40)	31(41)	12 (36)	0.63
Living donor‐recipient (%)	33 (31)	21 (28)	12 (36)	0.39
Pre‐emptive transplant (%)	22 (20)	15 (20)	7 (21)	0.89
End cPRA > 20% (%)	48 (44)	33 (44)	15 (46)	0.89
HLA mismatches (of 12)	7.7 ± 2.5	7.7 ± 2.6	7.6 ± 2.3	0.83
Pre‐transplant DSA present (%)	36 (33)	24 (32)	12 (36)	0.66
Kidney donor profile index %	42.6 ± 26.7	40.6 ± 25.8	47.8 ± 28.9	0.29
Induction (%)				0.61
Basiliximab	39 (36)	30 (40)	9 (27)	
Anti‐thymocyte globulin	55 (51)	36 (48)	19 (58)	
Alemtuzumab	14 (13)	9 (12)	5 (15)	
Delayed graft function (%)	14 (13)	5 (7)	9 (27)	0.004

Abbreviations: BMI, body mass index; cPRA, calculated panel reactive antibody; ESKD, end‐stage kidney disease; HLA, human leukocyte antigen.

The mean interval from transplant to the first biopsy with diagnosis of MVI ≥ 2 was 22.6 ± 24.7 months in the entire cohort (Table 2). At the time of the first biopsy, compared to the MVI (+) group, the MVI (−) group had significantly lower HLA DSA sum mean fluorescent intensity (MFI), lower g, ptc, MVI, and C4d scores. There were no significant differences in the kidney graft function or the management of AMR. Similar findings were noted at the time of the second biopsy (Table 2). Similar to the first and second biopsies, the sum MFI was significantly lower in the MVI (−) group. As expected, MVI scores were significantly lower in this group. Also, the transplant glomerulopathy (cg) score was significantly lower in this group.

**TABLE 2:** Biopsies and laboratory data.

	First biopsy				
		Total	Persistent MVI	Resolution of MVI	*p*
The mean interval from transplant to the first biopsy (mo)		22.6 ± 24.7	21.8 ± 23.2	24.3 ± 28.1	0.63
Maintenance immunosuppressive at the time of the biopsy					
Tacrolimus/mycophenolic acid/steroid (%)		83 (77)	55 (73)	28 (85)	0.42
Calcineurin inhibitor‐based (%)		105 (97)	73 (97)	32 (97)	0.92
DSA	Present (%)	69 (64)	49 (65)	20 (61)	0.64
	Against:				0.22
	Class I	11 (16)	6 (12)	5 (25)	
	Class II	31 (45)	21 (43)	10 (50)	
	Both	27 (39)	22 (45)	5 (25)	
	Mean sum MFI (among those with DSA)	12 291 ± 14 626	15 233 ± 15 853	5083 ± 7331	0.008
Graft function	Serum creatinine (mg/dL)	1.92 ± 1.22	1.82 ± 0.86	2.15 ± 1.80	0.19
	eGFR (mL/min/1.73 m^2^)	47.5 ± 21.1	47.3 ± 19.5	47.9 ± 24.6	0.88
Banff scores	g	1.56 ± 0.88	1.69 ± 0.91	1.27 ± 0.76	0.02
	ptc	1.26 ± 0.72	1.39 ± 0.77	0.97 ± 0.47	0.005
	MVI	2.82 ± 1.20	3.08 ± 1.26	2.24 ± 0.79	<0.001
	C4d	0.91 ± 1.30	1.13 ± 1.36	0.39 ± 0.99	0.006
	cg	0.32 ± 0.71	0.36 ± 0.75	0.24 ± 0.61	0.43
	cg+cv+ct+ci	2.09 ± 2.15	2.1 ± 2.1	2.0 ± 2.4	0.77
Rx of rejection	IVIG + steroid only	38 (35)	24 (32)	14 (42)	0.61
	IVIG + steroid + rituximab only	38 (35)	27 (36)	11 (33)	
	Addition of plasmapheresis	9 (9)	8 (11)	1 (6)	
	Addition of anti‐thymocyte globulin	8 (7)	7 (9)	1 (3)	
	Other (IVIG only, steroid only, etc.)	14 (13)	9 (12)	5 (15)	
The mean interval from transplant to the second biopsy (mo)		25.7 ± 24.9	25.1 ± 23.4	27.1 ± 28.4	0.70
The mean interval between first and second biopsies (mo)		3.2 ± 1.7	3.3 ± 1.9	2.8 ± 1.1	0.16
Maintenance immunosuppressive at the time of the biopsy					
Tacrolimus/MPA/steroid (%)		89 (82)	62 (83)	27 (82)	0.34
Calcineurin inhibitor‐based (%)		107 (99)	75 (100)	32 (97)	0.13
Quadruple immunos (%)		2 (2)	2 (3)	0	0.35
DSA	Present (%)	57 (53)	42 (56)	15 (45)	0.31
	Against:				0.71
	Class I	12 (21)	8 (19)	4 (27)	
	Class II	34 (60)	25 (60)	9 (60)	
	Both	11 (19)	9 (21)	2 (13)	
	Mean sum MFI (among those with DSA)	7626 ± 9323	9914 ± 10202	3364 ± 3919	0.04
Graft function	Serum creatinine (mg/dL)	1.75 ± 0.89	1.72 ± 0.60	1.82 ± 1.34	0.56
	eGFR (mL/min/1.73 m^2^)	48.8 ± 20.3	48.2 ± 18.8	50.3 ± 23.4	0.61
Banff scores	g	1.52 ± 0.84	1.69 ± 0.85	1.12 ± 0.65	<0.001
	ptc	1.16 ± 0.67	1.28 ± 0.65	0.87 ± 0.65	0.004
	MVI	2.68 ± 1.16	2.97 ± 1.15	2.0 ± 0.87	<0.001
	C4d	0.63 ± 1.13	0.75 ± 1.20	0.36 ± 0.92	0.11
	cg	0.28 ± 0.62	0.33 ± 0.70	0.15 ± 0.36	0.16
	cg+cv+ct+ci	2.48 ± 2.04	2.49 ± 2.09	2.45 ± 1.95	0.93
Rx of rejection	IVIG + steroid only	51 (47)	33 (44)	18 (55)	0.80
	IVIG + steroid + rituximab only	22 (20)	16 (21)	6 (18)	
	Addition of plasmapheresis	4 (4)	3 (4)	1 (3)	
	Addition of anti‐thymocyte globulin	2 (2)	1 (1)	1 (3)	
	Other (IVIG only, steroid only)	29 (27)	22 (29)	7 (21)	
The mean interval from transplant to the third biopsy (mo)		30.6 ± 25.1	29.8 ± 23.6	32.5 ± 28.6	0.61
The mean interval between the second and third biopsies (mo)		4.9 ± 3.9	4.7 ± 3.4	5.3 ± 4.9	0.44
Maintenance immunosuppressive at the time of the biopsy					
Tacrolimus/MPA/steroid (%)		87 (81)	60 (80)	27 (82)	0.30
Calcineurin inhibitor‐based (%)		107 (99)	75 (100)	32 (97)	0.13
Quadruple immunosuppressant (%)		6 (6)	5 (6)	1 (3)	0.47
DSA	Present (%)	52 (48)	40 (53)	12 (36)	0.11
	Against:				0.19
	Class I	12 (23)	9 (23)	3 (25)	
	Class II	31 (60)	22 (55)	9 (75)	
	Both	9 (17)	9 (23)	0	
	Mean sum MFI (among those with DSA)	4622 ± 10 793	6132 ± 12 409	739 ± 1004	0.05
Graft function	Serum creatinine (mg/dL)	1.87 ± 1.14	1.87 ± 1.08	1.86 ± 1.28	0.95
	eGFR (mL/min/1.73 m^2^)	48.1 ± 21.5	47.6 ± 20.2	49.2 ± 24.4	0.74
Banff scores	g	1.21 ± 0.99	1.71 ± 0.75	0.09 ± 0.29	<0.001
	ptc	0.95 ± 0.77	1.29 ± 0.63	0.18 ± 0.39	<0.001
	MVI	2.17 ± 1.54	3.0 ± 1.03	0.27 ± 0.45	<0.001
	C4d	0.63 ± 1.1	0.63 ± 1.1	−	
	cg	0.58 ± 0.88	0.75 ± 0.92	0.21 ± 0.65	0.003
	cg+cv+ct+ci	3.20 ± 2.02	3.21 ± 2.07	3.18 ± 1.94	0.94
Rx of rejection	IVIG + steroid only	25 (23)	22 (29)	3 (9)	<0.001
	IVIG + steroid + rituximab only	14 (13)	14 (19)	0	
	No active treatment	43 (40)	14 (19)	29 (88)	
	Other (IVIG only, steroid only)	26 (24)	25 (32)	1 (3)	
The median interval from transplant to last follow‐up (IQR, mo)		65.3 (46.9–91.5)	63.2 (46.2–89.2)	68.4 (49.3–95.5)	0.55
The median interval from first biopsy to last follow‐up (IQR, mo)		44.3 (31.0–60.4)	45.6 (30.8–58.9)	43.3 (31.7–66.2)	1.0
The median interval from third biopsy to last follow‐up (IQR, mo)		36.6 (22.4–53)	37.0 (20.3–52.2)	34.9 (24.2–54.5)	0.90
Total no. of biopsies during the study period		4.0 (3.0–5.0)	4.0 (3.0–5.0)	3.0 (3.0–4.0)	0.06
Graft function among those with graft survival	Serum creatinine (mg/dL)	1.81 ± 0.83	1.93 ± 0.77	1.55 ± 0.93	0.10
	eGFR (mL/min/1.73 m^2^)	49.7 ± 25.5	45.4± 20.4	59.1 ± 24.5	0.03
Graft failure (%)	Uncensored	48 (44)	34(45)	14 (42)	0.78
	Death censored	30 (28)	23 (31)	7 (21)	0.31

Abbreviations: cg, transplant glomerulopathy; ci, interstitial fibrosis; ct, tubular atrophy; cv, vascular fibrosis intimal thickening; DSA, donor‐specific antibody; eGFR, estimated glomerular filtration rate; g, glomerulitis; IVIG, Intravenous immunoglobulin; MFI, mean fluorescent intensity; MVI, microvascular inflammation; ptc, peritubular capillaritis.

The mean interval from transplant to the last follow‐up among the entire cohort was 69.9 ± 29.9 months (Table 2). At the time of the last follow‐up, there were no significant differences in the total number of biopsies done or the proportion of GF or DCGF between the groups. The MVI (−) patients with graft survival had significantly better eGFR at the last follow‐up at 59.1 ± 24.5 mL/min/1.73 m^2^ compared to 45.4 ± 20.4 mL/min/1.73 m^2^ (*p* = 0.03) in the MVI (+) group.

In Cox regression analysis, none of the commonly assessed baseline characteristics, Banff scores, or DSA status at the first or second biopsy were associated with persistent MVI on the third biopsy (Table 3). Also, in Cox regression analysis, from the first biopsy, after adjusting for various characteristics, persistent MVI on third biopsy was not associated with increased or decreased risk for GF (aHR: 0.55, 95% CI: 0.23–1.29; *p* = 0.17) (Table 4). The only factor associated with decreased risk for GF was use of depleting induction immunosuppression (aHR: 0.37, 95% CI: 0.16–0.86; *p* = 0.02). A factor associated with increased risk for GF was use of a quadruple immunosuppressive agent before the third biopsy (aHR: 5.19, 95% CI: 1.63–16.52; *p* = 0.005). Similar outcome results were found when assessing outcomes from the third biopsy to the last follow‐up interval (data not shown). Also, similar outcomes were found when assessing risk for DCGF (data not shown).

**TABLE 3:** Risk factors of having persistent MVI on the third biopsy.

Variables	Univariate			Multivariate		
	HR	95% CI	*p*	HR	95% CI	*p*
Age/yr	1.01	0.99–1.02	0.40			
Male	0.79	0.48–1.32	0.38			
Cause of ESKD: Diabetes mellitus vs. other	1.46	0.82–2.57	0.19			
Previous transplant	1.54	0.97–2.45	0.06	1.34	0.68–2.63	0.39
End cPRA > 20%	1.49	0.94–2.35	0.09	1.08	0.54–2.17	0.82
Pre‐transplant DSA present	1.44	0.88–2.34	0.15			
HLA mismatch (per)	1.02	0.94–1.13	0.54			
Kidney donor profile index	0.99	0.98–1.01	0.79			
Depleting induction	1.50	0.93–2.42	0.09	1.48	0.88–2.47	0.14
Delayed graft function	0.47	0.19–1.18	0.10	0.45	0.18–1.13	0.09
DSA present during 1st biopsy	0.88	0.55–1.42	0.61			
Sum DSA MFI at 1st biopsy	1.0	10–1.0	0.27			
1st biopsy MVI	1.13	0.93–1.38	0.22			
1st biopsy cg	0.80	0.59–1.08	0.15			
DSA present during 2nd biopsy	0.90	0.57–1.42	0.65			
Sum DSA MFI at 2nd biopsy	1.0	1.0–1.0	0.60			
2nd biopsy MVI	1.05	0.86–1.27	0.65			
2nd biopsy cg	0.81	0.56–1.18	0.27			
Quadruple immunosuppresion before 3rd biopsy	0.84	0.34–2.11	0.71			

Abbreviations: cg, transplant glomerulopathy; cPRA, calculated panel reactive antibody; DSA, donor‐specific antibody; ESKD, end‐stage kidney disease; HLA, human leukocyte antigen; MFI, mean fluorescent intensity; MVI, microvascular inflammation.

**TABLE 4:** Risk factors for uncensored graft failure from 1st biopsy to the last follow‐up.

Variables	Univariate			Multivariate		
	HR	95% CI	*p*	HR	95% CI	*p*
Age/year	1.02	0.99–1.04	0.09	0.99	0.96–1.03	0.73
Male	1.15	0.61–2.19	0.65			
Cause of ESKD: Diabetes mellitus vs. other	1.88	1.01–3.51	0.04	0.55	0.20–1.50	0.24
Previous transplant	0.56	0.30–1.03	0.06	0.98	0.44–2.21	0.96
End cPRA > 20%	0.75	0.42–1.34	0.33			
Pre‐transplant DSA present	0.91	0.49–1.68	0.77			
HLA mismatch	1.11	0.98–1.25	0.09	1.15	0.95–1.40	0.15
Kidney donor profile index	1.01	0.99–1.02	0.10	1.01	0.99–1.03	0.16
Depleting induction	0.58	0.33–1.02	0.06	0.37	0.16–0.86	0.02
Delayed graft function	1.73	0.86–3.51	0.12			
DSA present during 1st biopsy	1.60	0.85–3.03	0.15			
1st biopsy MVI	0.93	0.74–1.18	0.56			
1st biopsy cg	1.19	0.85–1.69	0.30			
DSA present during 2nd biopsy	1.23	0.70–2.19	0.47			
2nd biopsy MVI	0.91	0.71–1.15	0.42			
2nd biopsy cg	1.23	0.85–1.77	0.28			
Quadruple immunos before 3rd biopsy	4.06	1.58–10.42	0.003	5.19	1.63–16.52	0.005
Persistent MVI on 3rd biopsy	1.06	0.57–1.98	0.86	0.55	0.23–1.29	0.17

Abbreviations: cg, transplant glomerulopathy; cPRA, calculated panel reactive antibody; DSA, donor‐specific antibody; ESKD, end‐stage kidney disease; HLA, human leukocyte antigen; MFI, mean fluorescent intensity; MVI, microvascular inflammation.

**FIGURE 1:**
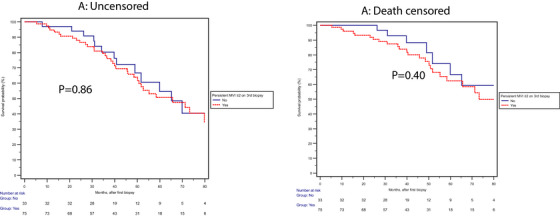
No significant differences in the uncensored (*p* = 0.86) or death‐censored *p* = 0.40) graft failure based on MVI (+) or MVI (−) on the third biopsy from the time of the diagnosis of AMR.

**FIGURE 2:**
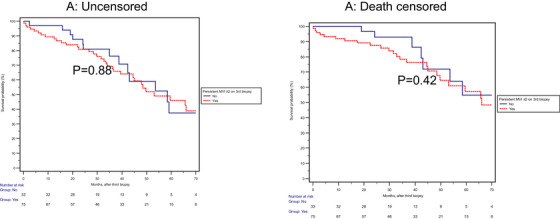
No significant differences in the uncensored (*p* = 0.88) or death‐censored *p* = 0.42) graft failure based on MVI (+) or MVI (−) on the third biopsy after the third biopsy follow‐up.

These findings were further confirmed by unadjusted Kaplan‐Meier survival analysis from the first biopsy for GF or DCGF (Figure 1A,B) or the third biopsy (Figure 2A,B).

Of 108 recipients with MVI ≥ 2 on the first biopsy, 32 (30%) had isolated MVI ≥ 2, without HLA‐DSA or C4d present; while the remaining 76 (70%), had either HLA‐DSA or C4d present to fulfill the criteria for AMR diagnosis on the first biopsy. Of these, 32 recipients with isolated MVI ≥ 2, without HLA DSA or C4d present, only 7 (22%) had cg ≥ 1, indicating features of chronic AMR. On the subsequent biopsies, at the second biopsy, 9 (28%) had cg ≥ 1, and 11 (34%) had cg ≥ 1 in the third biopsy. Of the remaining 76 recipients who met the criteria for AMR or probable AMR, 16 (21%) had cg ≥ 1. On the subsequent biopsies, at the second biopsy, 23 (30%) had cg ≥ 1, and 30 (39%) had cg ≥ 1 in the third biopsy. At the time of last follow‐up, 12 (38%) of 32 with isolated MVI ≥ 2 had GF, compared to 36 (47%) of 76 (*p* = 0.35), who fulfilled the criteria for AMR. Similarly, 7 (23%) with isolated MVI ≥ 2 had DCGF, compared to 23 (30%) (*p* = 0.38), who fulfilled the criteria for AMR.

Also, of 108 recipients with MVI ≥ 2 on the second biopsy, 41 (38%) had isolated MVI ≥ 2, without HLA‐DSA or C4d present; while the remaining 67 (62%), had either HLA‐DSA or C4d present to fulfil the criteria for AMR diagnosis on the second biopsy. Of these, 41 recipients with MVI ≥ 2, without HLA DSA or C4d present, only 12 (29%) had cg ≥ 1, indicating features of chronic AMR. On the subsequent biopsy, at the third biopsy, 15 (37%) had cg ≥ 1. Of the remaining 67 recipients who met the criteria for AMR or probable AMR, 10 (15%) had cg ≥ 1. On the subsequent biopsy, at the third biopsy, 26 (39%) had cg ≥ 1. At the time of last follow‐up, 16 (39%) of 41 with isolated MVI ≥ 2 had GF, compared to 32 (48%) of 67 (*p* = 0.38), who fulfilled the criteria for AMR. Similarly, 10 (24%) with isolated MVI ≥ 2 had DCGF, compared to 20 (30%) (*p* = 0.54), who fulfilled the criteria for AMR.

Of 75 recipients with persistent MVI ≥ 2 on the third biopsy, 30 (40%) had isolated MVI ≥ 2, without HLA‐DSA or C4d present; while the remaining 45 (60%), had either HLA‐DSA or C4d present to fulfil the criteria for AMR diagnosis on the third biopsy. At the time of last follow‐up, 13 (43%) of 30 with isolated MVI ≥ 2 had GF, compared to 21 (47%) of 45 (*p* = 0.78), who fulfilled the criteria for AMR. Similarly, 10 (33%) with isolated MVI ≥ 2 had DCGF, compared to 13 (29%) (*p* = 0.68), who fulfilled the criteria for AMR.

## 4 Discussion

In this large cohort of 108 KTRs with persistent MVI despite treatment, we found that only 31% had resolution of MVI, while the remaining 69 % had persistent MVI even after the two rounds of treatment for rejection. Also, outcomes were similar among those with isolated MVI ≥ 2, or those who fulfilled the criteria of AMR. Almost half of the kidney allografts failed just over 3 years from the third biopsies among those with previous MVI ≥ 2. The lack of difference in graft outcomes between the MVI ≥ 2 patients who were MVI (+) versus MVI (−) on the third biopsy suggests that subsequent response to AMR treatment is less important for prognosis than the initial development of AMR. Quadruple maintenance immunosuppression with the addition of belatacept before the third biopsy was associated with increased risk for graft failure, which could be related to treatment futility among a highly selected group with underlying severe Banff scores, rather than treatment itself. This reinforces the importance of the prevention of rejection, along with aggressive treatment at the beginning.

Persistent MVI after the management of AMR is not unusual. In one study among 90 KTRs with AMR and follow‐up biopsy within a median of 2 months, 50% had MVI ≥ 2 despite aggressive treatment for AMR. [9] Similar to our study, there were also no significant histological differences in the biopsy at AMR diagnosis between patients with and without the persistence of microvascular inflammation, except for a greater severity of MVI in patients with persistent MVI. And death censored graft survival was significantly lower in those patients with persistent MVI [9]. In a study by Koenig et al., graft survival among groups based on the presence of MVI, DSAs, and C3d was found to be equivalent between the MVI+DSA+C3d− and MVI+DSA−C3d− groups. Moreover, the graft survival of these two groups was better than that of the MVI+DSA+C3d+ group but significantly worse than that of the MVI−DSA− group, suggesting a deleterious impact of MVI on graft survival [13]. All these findings support the aggressive management of MVI at the time of first diagnosis, as does a recent study among pediatric KTRs [14]. However, in the current study, we noticed that those with persistent MVI after the second biopsy would have similar detrimental outcomes whether MVI persisted or resolved. Also, there were no commonly assessed variables that predicted resolution or persistent MVI.

Anti‐HLA DSAs are an important biomarker for predicting graft injury and failure [15]. However, in one study from our group, we reported that just the presence of anti‐HLA DSA in the absence of graft rejection was not associated with risk for graft failure [16]. The presence of MVI, even in the absence of anti‐HLA DSA, was associated with similar detrimental outcomes as having anti‐HLA DSA [17]. Another important component for the diagnosis of AMR is C4d; similar to anti‐HLA DSA, C4d‐positive or negative AMR had comparable poor graft outcomes [18].

Given these findings, and the significantly higher rate of graft failure after AMR, close monitoring with follow‐up biopsy is recommended. Importantly, the trend of serum creatinine does not predict follow‐up biopsy findings among recipients with AMR [19]. However, recently, donor‐derived cell‐free DNA (dd‐cfDNA) has been getting more attention for the early diagnosis of rejection, along with monitoring for the response to the treatment of rejection. The significant changes of dd‐cfDNA from baseline observed before and after treatment of rejection could be an added tool for monitoring treatment response for AMR [20].

Our observations have the limitations inherent to this type of study. As a single‐center, prospective, non‐randomized study, it may not be possible to generalize our results to other centers. There could be some selection bias in the utilization of treatment options, but there were no significant differences in the basic demographics or initial histopathological findings. Further, we included only recipients who had graft survival and had at least two follow‐up biopsies after initial AMR diagnosis. However, to our best knowledge, this is the largest reported series comparing various features of persistent MVI ≥ 2, based on the two follow‐up biopsies. Two strengths in our study were the ability to provide granular data, and the majority of biopsies were read by a single renal pathologist, minimizing interobserver bias. In summary, management of AMR is complex, and the response rate to the treatment is suboptimal. The lack of difference in graft outcomes between the AMR patients on the third biopsy suggests that subsequent response to AMR treatment is less important for prognosis than the initial development of AMR.

## Author Contributions

**Sandesh Parajuli**: manuscript preparation, data collection, design, analysis, writing. **Adam Bregman**: data collection, editing. **Emily E. Zona**: data collection, editing. **Megan Sokup**: data collection, editing. **Neetika Garg**: editing. **Weixiong Zhong**: editing. **Didier Mandelbrot**: original idea, writing, editing.

## Funding

This study was supported by an unrestricted research grant from the Virginia Lee Cook Foundation to Dr. Mandelbrot.

## Conflicts of Interest

The authors declare no conflicts of interest.

## Data Availability

The data that supports the findings of this study are available from the corresponding author, [SP], upon reasonable request.
